# Challenging Diagnostic Dilemma: Mesenteric Desmoid Tumor Masquerading as Perforated Peritonitis

**DOI:** 10.7759/cureus.42946

**Published:** 2023-08-04

**Authors:** Siddharth Sankar Das, Sahil Navlani, Arfan Alawa, Ferial M Abbas, Lakshmiah G Raman, Akshata Mestha

**Affiliations:** 1 General Surgery, Dubai Hospital, Dubai, ARE; 2 Medical School, Dubai Academic Health Corporation, Dubai, ARE; 3 General Surgery, Dubai Health Authority, Dubai, ARE; 4 Pathology, Dubai Hospital, Dubai, ARE

**Keywords:** gastrointestinal stromal tumor, rare abdominal tumor, aggressive fibromatosis, soft tissue neoplasm, desmoid fibromatosis

## Abstract

Desmoid fibromatosis is a rare benign neoplasm of the soft tissue. Primary desmoid neoplasms rarely occur in the small bowel and are primarily found in patients with a previous abdominal surgery or irradiation history. They are challenging to diagnose at the time of presentation due to a lower incidence and their non-specific presentation making it difficult to distinguish from other intra-abdominal neoplasms, such as gastrointestinal stromal tumors (GISTs), which may present with similar symptoms. We like to present a case of a 34-year-old male with a four-day history of abdominal pain with worsening severity and one episode of non-bloody vomiting. Physical examination was significant for generalized abdominal tenderness with positive rebound and board-like rigidity. A computed tomography (CT) scan of the abdomen showed the presence of a lower abdominal mass of unknown etiology with free air foci and free intraperitoneal fluid either due to rupture of the suspicious mass or secondary to infection by an air-producing organism. The patient was immediately taken for emergency surgery, the tumor was resected successfully, and a specimen collected was sent for histopathology, which came out to be a desmoid tumor. We aim to highlight the importance of keeping a broad differential diagnosis in a patient with acute abdomen and symptoms of peritonitis.

## Introduction

Desmoid tumors are locally aggressive but benign soft tissue tumors originating from the mesenchymal cells with a high recurrence rate but without the propensity to metastasize [1]. The estimated incidence rate of desmoid tumors is two to four cases per million in the general population. It occurs more commonly in females and ages between 15 and 60 [2]. It is found to occur in the intra-abdominal soft tissues with a reported incidence of 8% [3]. Studies have found a strong association between familial adenomatous polyposis and desmoid neoplasms [1,4]. An association has also been found between desmoid neoplasms and Gardner syndrome [5].

## Case presentation

A 34-year-old male presented to the emergency department with abdominal pain that started in the periumbilical area and later migrated to the right iliac fossa with one episode of non-bloody vomiting and a fever of four days duration. Past surgical history was significant for an open inguinal hernia repair done seven years back. On physical examination, the patient was febrile, tachycardic, and ill-appearing. Abdominal examination was significant for generalized abdomen tenderness with rebound and board-like rigidity. The rest of the physical examination was otherwise unremarkable. A working diagnosis of perforated appendicitis was made.

**Figure 1:**
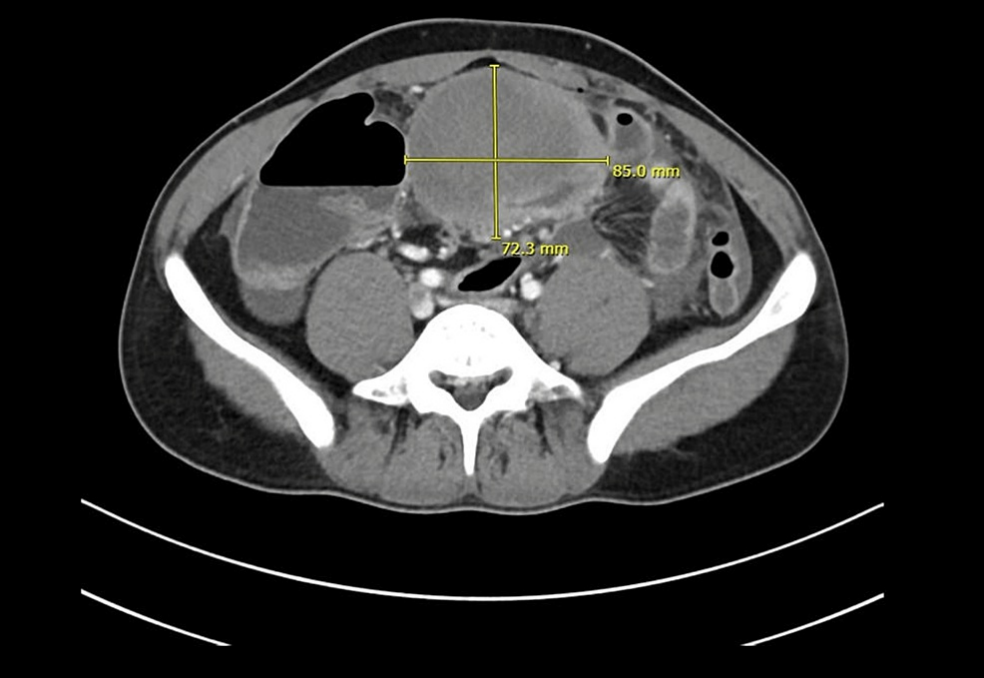
CT abdomen axial view showing the presence of a large mass in the lower abdomen CT, computed tomography

**Figure 2:**
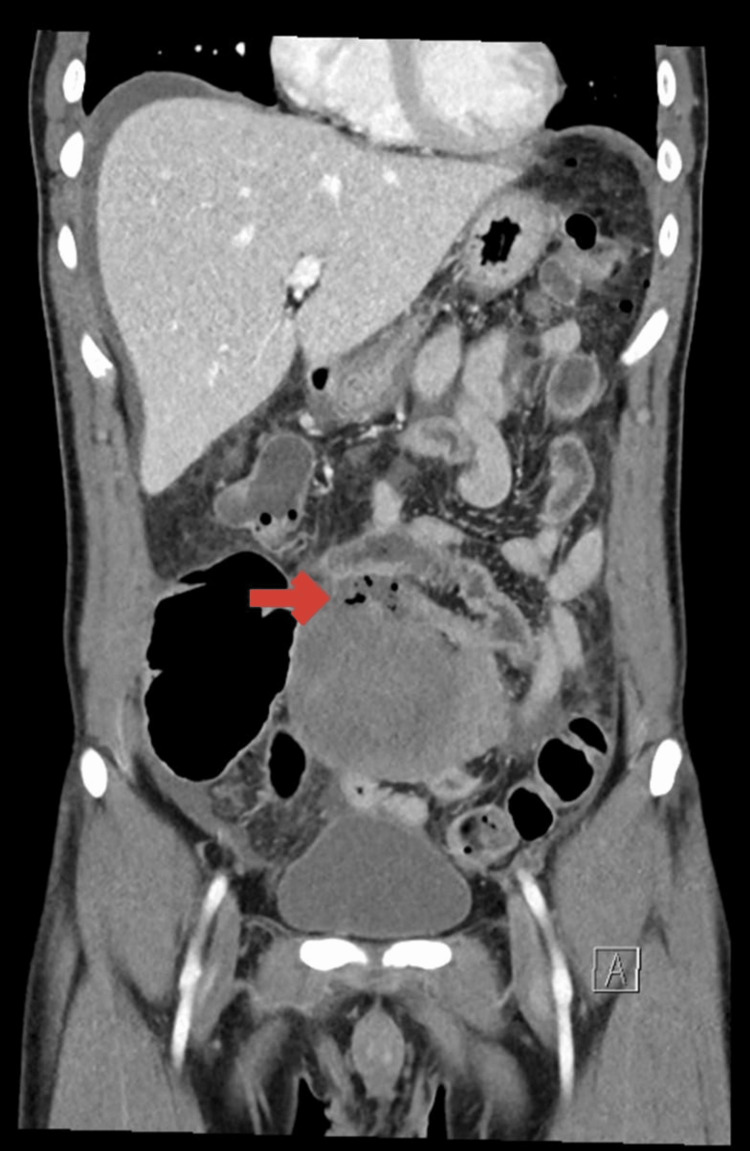
CT abdomen coronal view showing the presence of a large mass with the presence of multiple air foci CT, computed tomography

An erect X-ray of the chest and abdomen was done to rule out perforated peptic ulcer disease. The X-ray abdomen showed the presence of few air-fluid levels. Then the decision to proceed with a CT abdomen was made, which showed a large mass with a hypodense center and a marginal enhanced shaggy wall seen at the center of the lower abdomen, inseparable from the nearby small bowel loops (Figure 1). Also, multiple air foci were present in the periphery of the mass, suggesting that the mass arose either from the small bowel or its serosal covering (Figure 2). Mild to moderate free peritoneal ascites were noted with regional secondary peritonitis and congested mesenteric vessels. There was perifocal multiple lymph node enlargement, the largest measuring about 15 × 10 mm in diameter. Based on the radiological findings, there was a differential diagnosis of acute abscess or abscess on top of a pre-existing neoplastic mass, possibly carcinoid or gastrointestinal stromal tumor (GIST).

**Figure 3:**
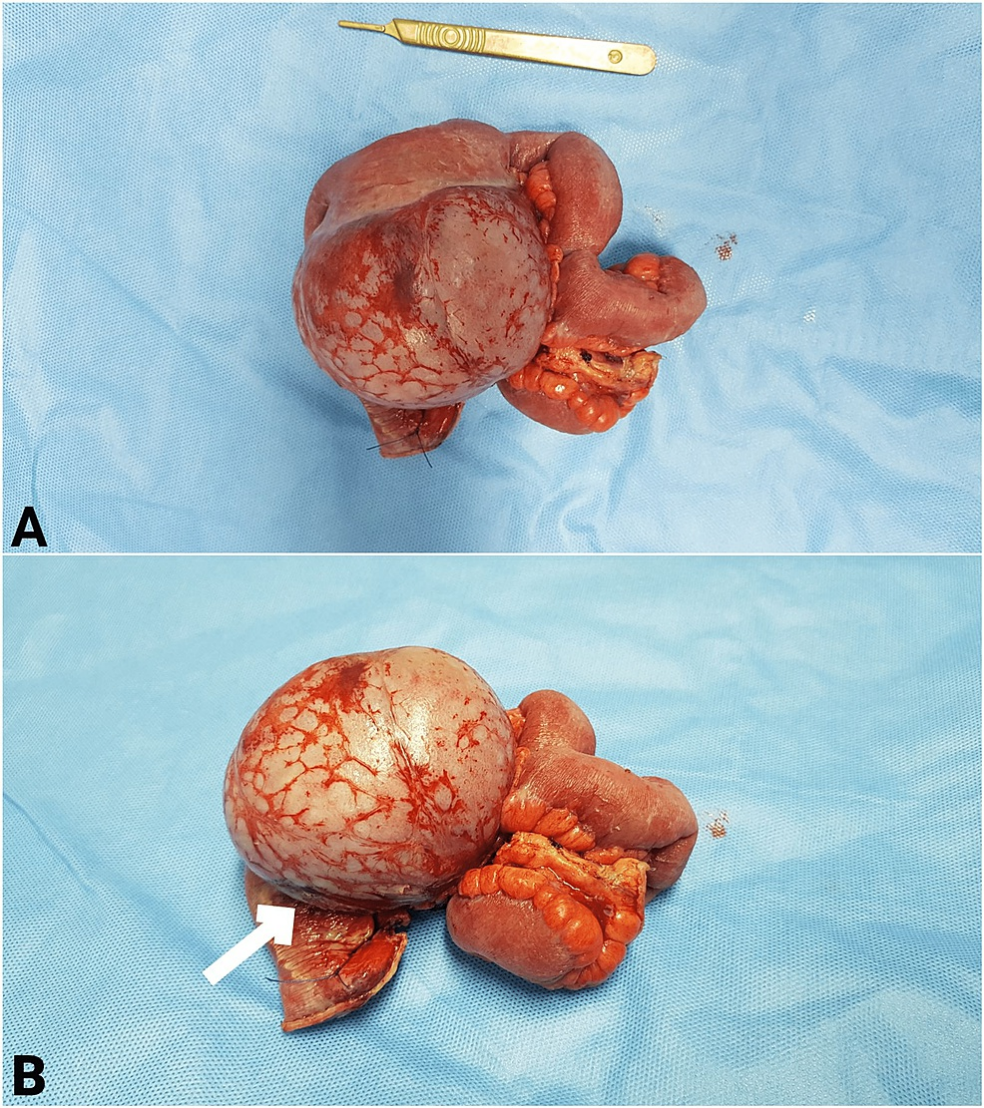
Gross anatomy of the collected specimen (A) Well-circumscribed mass (B) Site of perforation of the mass

The patient underwent diagnostic laparoscopy, which showed the presence of a large lower intra-abdominal mass arising close to the distal ileal mesentery root with the involvement of a segment of the ileum loop. The procedure was converted to laparotomy, which showed a sizable perforated mass of around 10 cm, arising close to the root of mesentery of the distal ileum (Figure 3A), involving the corresponding adjacent ileal loop. The ileal segment was densely adherent to the tumor and could not be separated from the mass. There was a small perforation in the capsule of the tumor adherent to the intestine segment, along with intestinal perforation due to necrosis (Figure 3B). The tumor was resected using a harmonic scalpel, from the root of the mesentery, along with the affected segment of the ileum. After resection of the whole tumor mass and affected segment of the intestine, the remaining distal ileum segment was only 8 cm from the terminal ileum in the vicinity of the ileocecal junction. Hence, limited cecectomy was also performed, and ileo-ascending anastomosis was performed using a linear stapler. The resected segment was sent for histopathology. A thorough abdominal lavage was done with warm normal saline. The abdomen was closed with two drains inserted, one in Morrison's pouch and the other in the pelvis. The drains had minimal output in the postoperative period.

**Figure 4:**
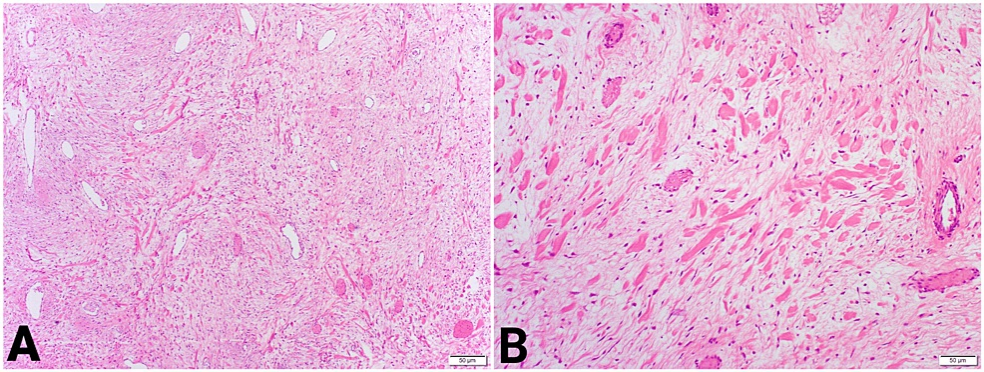
Hematoxylin and eosin (H&E) staining (A) 100× magnification: broad, sweeping fascicles of uniform fibroblastic cells within a collagenous stroma (B) 200× magnification: hyalinized, keloidal-type distribution of collagen fibers

The peritoneal fluid was sent for microscopy, revealing scattered mesothelial cells in a background composed of neutrophils, histiocytes, lymphocytes, and occasional plasma cells. However, no component of malignant cells was seen from the sample obtained. The histology report of the collected sample showed an intestinal spindle cell tumor with capsular rupture, partial infarction, ischemic changes, and involvement of the mesenteric margin. The lateral margins of resection were free of the lesion. The pathological slides reported features suggestive of desmoid fibromatosis (Figures 4A, 4B).

**Figure 5:**
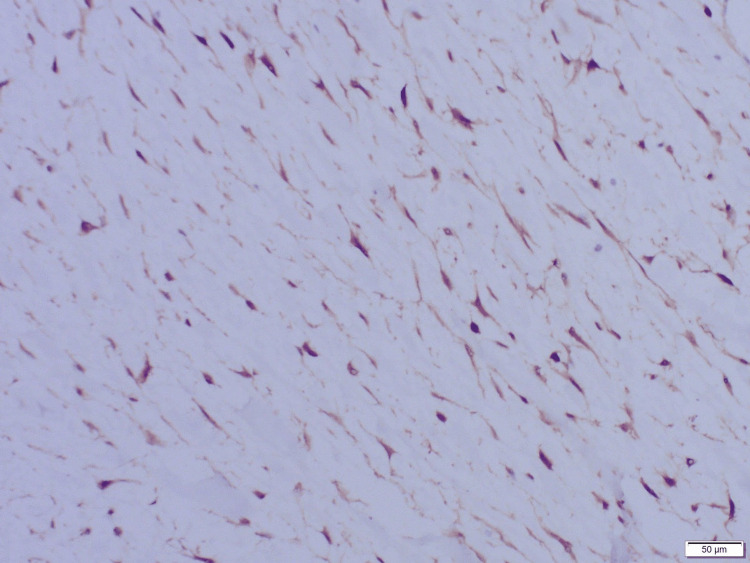
Immunohistochemical stain at 100× magnification showing nuclear positive for β-catenin

Immunohistochemical stains were performed and were strongly positive for β-catenin and vimentin (Figure 5). Other positive stains were smooth muscle actin (SMA), cyclin D1, and calretinin. These results were confirmatory for the presence of a dermoid neoplasm. The patient was stable without any active symptoms at the time of discharge.

## Discussion

Desmoid neoplasms are infrequent and comprise 0.03% of all neoplasms and 3% of soft tissue neoplasms in general [6]. Extra-abdominal desmoid fibromatosis is most frequently observed in the abdominal wall (50%) and the extra-abdominal soft tissues of the trunk or limbs (40%) [3]. On the other hand, intra-abdominal neoplasms are the least prevalent (8%) and mainly impact the mesentery of the small bowel, the ileocolic region, and the mesocolon [3]. Intra-abdominal desmoid tumors most commonly occur due to prior abdominal surgery [7].

Desmoid tumors are benign mesenchymal tumors that exhibit fibroblastic cells embedded in a stroma of collagen and spindle cells in histology. According to several studies, there is a higher incidence of desmoid tumors in female patients, especially those with a history of oral contraceptive pills and during gestation and postpartum, which is in contrast to the male patient in this case [8]. A prior history of abdominal wall trauma has been linked to the development of desmoids [9]. It has been suggested to occur possibly due to a molecular connection between the wound healing process and the formation of fibro-proliferative pathologies in the mesenchymal tissue [9]. This could be a possible etiology of the tumor development in this patient, who had a history of a prior laparotomy. Other etiologies that have been stated to be involved in the development of desmoid tumors include a history of irradiation, genetic mutations such as familial adenomatous polyposis and Gardner syndrome, and a positive family history of desmoid tumors [10-13].

It has also been reported to occur due to the intranuclear accumulation of β-catenin, which has been found to occur due to mutations in the Wnt/β-catenin gene. Immunostaining markers of β-catenin have, in recent times, immensely helped in detecting antigens that can assist in differentiating between desmoid neoplasms and other fibroblastic neoplasms that may have similar findings on gross appearance and histology [14]. Our patient’s histology was characteristic of a desmoid tumor, and his immunohistochemical stain was also strongly positive for β-catenin, which enabled us to reach the diagnosis.

Intra-abdominal tumors usually do not manifest with any symptoms in the early stages. As a result, the commonly encountered signs and symptoms of abdominal pain, vomiting, gastrointestinal bleeding, or a palpable mass are usually not detected until late in the course of the disease. The complications that may be commonly encountered due to intra-abdominal tumors include entero-cutaneous fistulae, intestinal perforation, intestinal hemorrhage, bowel obstruction, and ureteric obstruction [4]. Our patient had come in the late stage with an enlarged mass that had perforated; as a result, he presented as a case of acute abdomen with peritonitis.

Due to the uncertain etiology and life-threatening condition, a surgical approach for management had been planned in our case. However, research articles have found that different options can be employed to manage patients with desmoid tumors. Chemotherapy, radiotherapy, and antihormonal therapy with nonsteroidal anti-inflammatory drugs are successful. It is encouraged to manage such tumors meticulously by starting with less toxic medications and then escalating to more potent drugs if necessary. A “watch and wait strategy” is proposed for patients, and active intervention is only recommended in patients with a progressive course or acute symptoms. [15] On the other hand, research by Escobar et al. argues that complete resection is more favorable for a symptomatic tumor with well-defined microscopic margins [16]. However, one research has found a 35% risk of tumor recurrence after resection with negative margins [17]. Nevertheless, the mainstay of management in the past involved using low doses of chemotherapy. However, recent developments have found good responses to tyrosine kinase inhibitors like sorafenib and pazopanib [18].

## Conclusions

In view of the relative rarity of the incidence of the neoplasm intra-abdominally, there has been difficulty in establishing a standard plan of care. It may be challenging to differentiate from other neoplasms that present similarly, such as GIST; hence, there is a need for biopsy and further immuno-staining and histology. Desmoid fibromatosis may present with atypical signs and should be included when evaluating patients with peritonitis.
